# Supplementary figures and images for: A novel open-source raspberry Pi-based behavioral testing in zebrafish
Source: PLoS One. 2022 Dec 27;17(12):e0279550. doi: 10.1371/journal.pone.0279550 (PMC9794099; doi:10.1371/journal.pone.0279550)

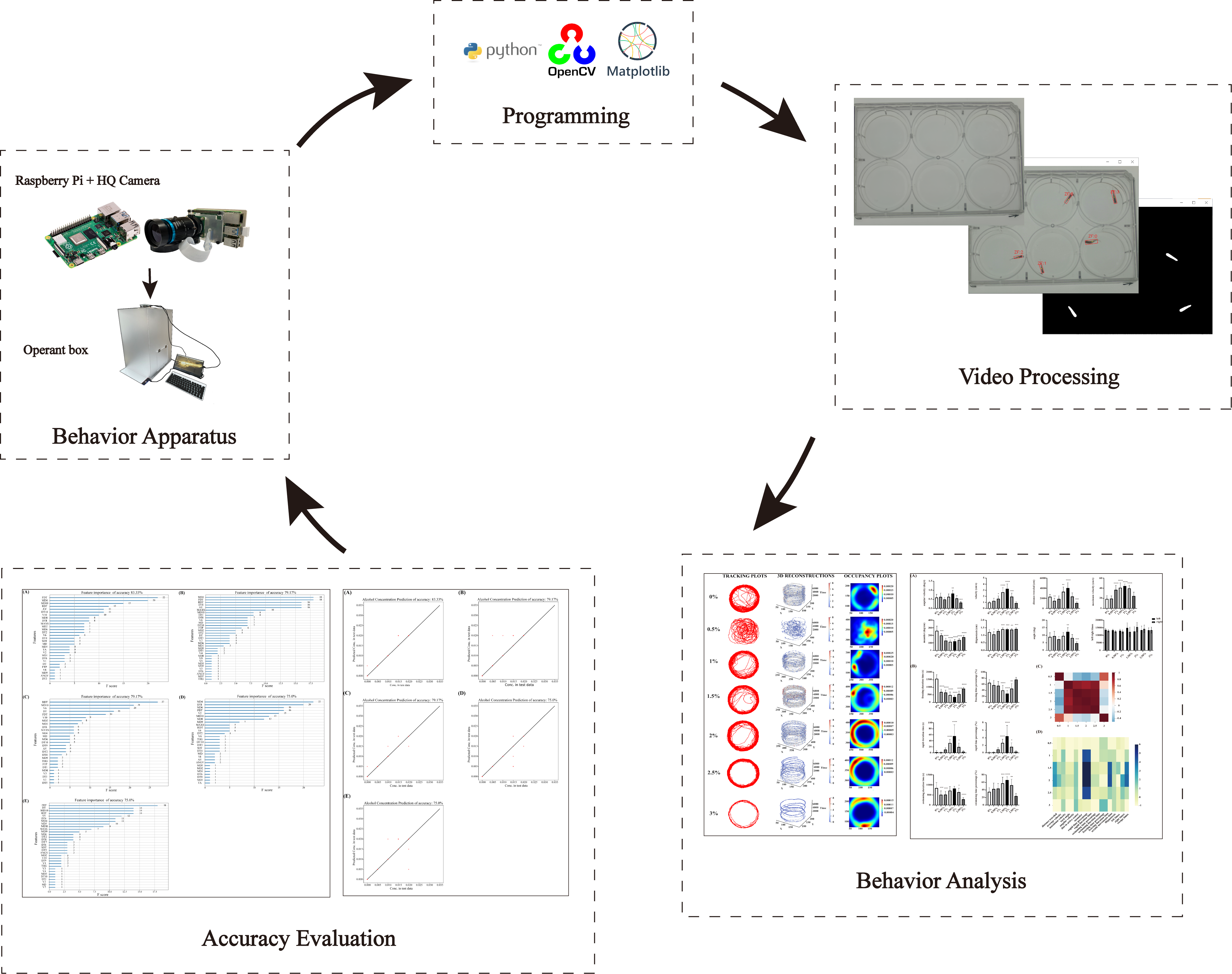

Supplement: S1 Graphical abstract — (TIF) [file pone.0279550.s005.tif]
