# Supplementary material for: A novel open-source raspberry Pi-based behavioral testing in zebrafish
Source: PLoS One. 2022 Dec 27;17(12):e0279550. doi: 10.1371/journal.pone.0279550 (PMC9794099; doi:10.1371/journal.pone.0279550)
Supplement: Attachment — Submitted filename: Response to Reviewers.docx [file pone.0279550.s006.docx]

**Response**

First of all, we thank the reviewers for your comments and suggestions on our manuscript “A novel open-source raspberry Pi-based behavioral testing in zebrafish”. Based on the constructive and helpful comments, we addressed the raised questions and revised our manuscript carefully. Some changes have been made in this revision and our responses to each point (in blue colors) are as follows:

Reviewer #1: Thank you for sharing your manuscript with me. The author designed a low-cost automatic zebrafish behavior detection device and software to measure zebrafish behavior parameters. The results show that the device designed by the author can be used to obtain video data and determine various behavioral parameters to reliably evaluate the behavioral performance of zebrafish. Generally speaking, the theme of the manuscript is very meaningful and necessary. But there are many problems that need to be corrected. These issues are listed below and may help improve the manuscript.

Q1. Figure 4 is not clear enough. The horizontal and vertical coordinates of each result cannot be seen clearly. Please replace the result map with a higher definition.

Response: Thank you for pointing out the weakness of our paper. According to your suggestion, we have modified the Figure 4 by changing it to a vector image (format: eps or tiff) with higher resolution. In addition, barplots for parameters were replaced by boxplot with individual data points jittered to allow fully and easy comprehension of the data.

Figure 4

Q2. What is the composition of the system water mentioned in line 91?

Response: Sorry for missing of details. This information was added to the paragraph as: “Zebrafish were maintained in 4 L tanks (20-30 fish per tank) with system water (dechlorinated tap water) at 28±2 ℃, pH 7.2-7.5 and conductivity 700 muS.”

Q3. The zebrafish of one month old is used in the experiment. Can you track and analyze the behavior of small fish several days old after fertilization or adults of three months old through this device and software?

Response: Thank you for proposing such an important question. We tracked and analyzed the behavioral videos (around 60 seconds) of 5 days postfertilization (dpf) and adults of three months old zebrafish through our device and software. Both three-month and five-day post-fertilization zebrafish could be identified. The 5 dpf zebrafish had a translucent and very small body, which made identification more difficult and required a higher resolution of the camera. Three-month-old zebrafish are easier to identify because of their relatively larger size and opaque body.

Tracking results of three-month-old zebrafish

Binarization results of video images of three-month-old zebrafish

Trajectory of three-month-old zebrafish

Tracking results of 5 days postfertilization zebrafish

Binarization results of video images of 5 days postfertilization zebrafish

Trajectory of 5 days postfertilization zebrafish

Q4. Line 163: regions of interest (ROIs) were defined to eliminate the false recognition caused by the mirror effect (fish reflection on the well-wall) of zebrafish in the multi-well plate and the possible influence of the edge of the multi-well plate. The region of interest here refers to which region?

Response: The region of interest (ROI) here refers to each well of multi-well plates. We use a python script to mark the position of each well of the multi-well plate on the extracted background image (Fig2. A), and then we use ‘select_circle_roi.py’ in ‘S1 codes/pre-processing’ to further determine the ROI. The image processing effect after adding ROI is shown below. The specific location information of the ROIs is collected once with the same camera and multi-well plate placement.

Mark the region of interests

The image processing results of adding the ROI

Reviewer #2: This study provides a system package for determining multiple behavioral parameters of zebrafish, a model organism in the behavioral tests. In addition, the authors used an alcohol exposure test to examine the validity of their method. The MS has some novelty, but the following issues must be clarified before being recommended for publication.

Q1 Tables: Three-line tables should be used, and footnotes should be used to indicate the meaning of abbreviations.

Response: Sorry for making this mistake. Done as suggested.

**Table 1**. Image processing thresholds

| Threshold | Value | Description |
| --- | --- | --- |
| Th_sp_ | 160 | Total frames for background extraction |
| Th_min_ | 60 | Minimum object area |
| Th_max_ | 800 | Maximum object area |

**Table 2.** Definition of behavioral endpoints

| Parameters | Formula | Description |
| --- | --- | --- |
| Distance (cm) | $\begin{matrix} \Delta s_{i}=\sqrt{(x_{i}-x_{i-1})^{2}+(y_{i}-y_{i-1})^{2}} \\ s=\sum_{i=1}^{t} \Delta s_{i} \end{matrix}$ | The total distance of zebrafish swimming within a certain time, namely the sum of the Euclidean distance of the zebrafish position in all consecutive frames. |
| Velocity (cm/s) | $v_{i}(t)=\frac{d\Delta s_{i}}{dt},i\in\left[1,n-1\right]$ | Distance of zebrafish swimming per unit time. |
| Maximum velocity (cm/s) | $\max\left\{v_{1},...,v_{i},...,v_{n-1}\right\}$ | A maximum speed of fish swimming within 20 min. |
| Angle (deg) | $ang=\arccos\left(\frac{dot(vect_{1},vect_{2})}{\left\vert vect_{1}\right\vert\left\vert vect_{2}\right\vert}\right)\times\frac{180}{\pi}$ | The rotation angle of zebrafish between consecutive frames. |
| thigmotaxis | $thig=\frac{time(wall)}{time(wall)+time(center)}$ | The time in the outer circle versus the total times. |

Table 2 (continued)

| Parameters | Formula | Description | |
| --- | --- | --- | --- |
| Meander (deg/cm) | $meander=\frac{ang}{\Delta s_{i}}$ | | The sum of rotation angles of zebrafish within unit distance. |
| Erratic movements | $v_{i}(t)>3,erratic+=1$ | | Times when the velocity change of zebrafish is greater than 3 cm/s. |
| Escape times | $\left\{\begin{matrix} v_{i}(t)>5 \\ ang>45^{\circ} \end{matrix}\right.,escape+=1$ | | Times when the velocity and angle of zebrafish change greatly at the same time. |
| Freezing duration time (s) | $\begin{matrix} 0<v_{i}(t)<1,times+=1 \\ duration\_time=\frac{times}{fps} \end{matrix}$ | | Duration time when the velocity of zebrafish is less than 1 cm/s. |
| Freezing time percentage (%) | $\begin{matrix} 0<v_{i}(t)<1,times+=1 \\ percentage=\frac{times}{n} \end{matrix}$ | | Percentage of the state when thevelocity of zebrafish is less than 1 cm/s. |
| Swimming duration time (s) | $\begin{matrix} 1<v_{i}(t)<10,times+=1 \\ duration\_time=\frac{times}{fps} \end{matrix}$ | | Duration time when the velocity of zebrafish is between 1-10 cm/s. |

Table 2 (continued)

| Parameters | Formula | Description |
| --- | --- | --- |
| Swimming time percentage (%) | $\begin{matrix} 1<v_{i}(t)<10,times+=1 \\ percentage=\frac{times}{n} \end{matrix}$ | Percentage of the state when the velocity of zebrafish is between 1-10 cm/s. |
| Rapid duration time (s) | $\begin{matrix} v_{i}(t)>10,times+=1 \\ duration\_time=\frac{times}{fps} \end{matrix}$ | Duration time when the velocity of zebrafish is large than 10 cm/s. |
| Rapid time percentage (%) | $\begin{matrix} v_{i}(t)>10,times+=1 \\ percentage=\frac{times}{n} \end{matrix}$ | Percentage of the state when the velocity of zebrafish is large than 10 cm/s. |
| Left/Right times | $\begin{matrix} \det=\det(vect_{1},vect_{2}) \\ \left\{\begin{matrix} \det<0,right+=1 \\ \det>0,left+=1 \end{matrix}\right. \end{matrix}$ | Times of left and right deflections in the process of zebrafish moving. |

**Table 3.** Automatic zebrafish recognition and tracking assessment

| Video dataset | Fish number | Truth | TP | FP | FN | P | R | F |
| --- | --- | --- | --- | --- | --- | --- | --- | --- |
| D1 | 6 | 29959 | 29878 | 14 | 67 | 0.9995 | 0.9978 | 0.9986 |
| D2 | 6 | 29958 | 29914 | 24 | 20 | 0.9992 | 0.9993 | 0.9993 |
| D3 | 6 | 29955 | 29955 | 0 | 0 | 1 | 1 | 1 |
| D4 | 6 | 29955 | 29914 | 0 | 41 | 1 | 0.9986 | 0.9993 |
| D5 | 6 | 29954 | 28956 | 20 | 978 | 0.99931 | 0.967328 | 0.983059 |
| D6 | 6 | 29955 | 29644 | 42 | 269 | 0.998585 | 0.991007 | 0.994782 |
| D7 | 6 | 29955 | 29872 | 42 | 41 | 0.998596 | 0.998629 | 0.998613 |

TP, true positive; FP, false positive; FN, false negative; P, precision; R, recall; F, F-score.

Q2 Statistical analysis method: Since behavioral data usually cannot satisfy normal distribution and Homogeneity, one-way ANOVA and unpaired t-test are not necessarily applicable. At this time, it is best to use the generalized linear model (GzLM) for analysis. See the references:

Persistent impact of amitriptyline on the behavior, brain neurotransmitter, and transcriptional profile of zebrafish (Danio rerio). Aquatic Toxicology, 2022, 245 10.1016/j.aquatox.2022.106129.

Impacts of chronic exposure to sublethal diazepam on behavioral traits of female and male zebrafish (Danio rerio). Ecotoxicology and Environmental Safety, 2021, 208 10.1016/j.ecoenv.2020.111747.

Response: Thank you for pointing out the weakness of our paper. The normality consumption of these parameters were assessed based on the Shapiro-Wilk normality test and visualized by QQplot. It was found that most of these parameters did not conform to a normal distribution, with p-values less than 0.05. In addition, parameters were not in linear relationship with alcohol concentrations (Figure 4). As suggested, the generalized linear model (GzLM) analysis was performed using a gaussian distribution and identity link function, considering alcohol concentration as categorical variable. Figure 4 in manuscript was thus also reorganized. In addition, barplots for parameters were replaced by boxplot with individual data points jittered to allow fully and easy comprehension of the data.

Gaussian distribution of parameters assessed by Shapiro-Wilk normality test and graphically by QQplot

Q3. The author should provide some videos to give readers an intuitive impression.

Response: Thank you for your valuable comments and advice. According to your suggestion, we have uploaded some videos as supporting information files, including the original video, the zebrafish identification and tracking process video, the mask after ROI operation video and the binarization process video after image processing. In order to comply with the uploaded file size regulations and for quick and easy viewing, only 50 seconds of the experimental video is captured here for reference.

Q4. The authors should also discuss and identify the innovations and advantages of their methods. For example, using their systems and commercial equipment to track and analyze the trajectory of the same subjects, compare their results, and then identify the advantages and disadvantages.

Response: Thank you for proposing such an important question. We used Cohen's Kappa to measure the agreement between the platform proposed in this study and the commercial platform. It was found that the agreement of most of the parameters was greater than 0.33. Among them, the agreement of parameters such as average velocity, rapid duration time and rapid time movement percentage reached 1, which indicates a fair agreement between the two platforms.

Comparison of parameters measured by Commercial and Current platform

Q5. Social behavior is the most difficult in fish behavioral analysis. Because when multiple individuals are located in the same container, it is easy to cause exchange between subjects (especially when behavioral trajectories cross each other), and it also involves complex interactions between subjects. I would like to know if there is an excellent way to solve the above problem with this methods.

Response: Thank you for proposing such an important problem. In this study, a multi-well plate was used as a container to hold multiple zebrafish and ensured only one fish was placed in each well, thus avoiding problems such as trajectories cross each other and targets exchange that may arise from multiple zebrafish in one space. In addition, we further exclude the possibility of target exchanging between zebrafish in adjacent wells due to their close proximity by determining the region of interest (ROI) and using a target matching algorithm.
